# Traffic accident duration prediction using text mining and ensemble learning on expressways

**DOI:** 10.1038/s41598-022-25988-4

**Published:** 2022-12-12

**Authors:** Jiaona Chen, Weijun Tao

**Affiliations:** 1 National Engineering Laboratory for Surface Transportation Weather Impacts Prevention, Broadvision Engineering Consultants, Kunming, 650200 China; 2 Xi’an Shiyou University School of Electronic Engineering, Xi’an 710065, China

**Keywords:** Civil engineering, Electrical and electronic engineering

## Abstract

Predicting traffic accident duration is necessary for ensuring traffic safety. Several attempts have been made to achieve high prediction accuracy, but researchers have not considered traffic accident text data in much detail. The limited text data of the first report on an incident describes the characteristics of an accident that are initially available. This paper uses text data fusing and ensemble learning algorithms to build a model to predict an accident’s duration, and a preprocessing scheme of accident duration text data is established. Next, the random forest (RF) algorithm is applied to select feature variables of text data related to the traffic incident duration. Last, a text feature vector is introduced to models such as decision tree, k nearest neighbor, support vector regression, random forest, Gradient Boosting Decision Tree, and Xtreme Gradient Boosting. Our results show that the improved RF model has good prediction accuracy with RMSE, MAPE and R^2^. From this, the textual factors important to determining the duration of the accident are identified. Further, we investigated that the cumulative importance of 60% is sufficient for traffic accident prediction using text data. These results provide insights into minimizing traffic congestion related to accidents and contribute to the input optimization in text prediction.

## Introduction

Road safety has become an urgent social concern considering the increased rate and uncertainty associated with traffic accidents. Sustainable transport systems are means of transport that have lower impact on the environment. However, traffic accidents are one of the negative social effects for the environment. Response time and the efficiency of handling incidents are coming into the public attention. Rapid recovery from traffic accidents is an important research issue for the sustainability of traffic systems. Predicting the duration of traffic accidents has important implications for traffic management and travelers. Long-term forecasting has become an important issue in road safety in recent years. With the development of traffic management and information requirements, a large amount of traffic accident data is collected by the traffic management systems, which include both structured and unstructured data. Structured data are features that can be quantified in a fixed format (e.g., a table). Unstructured data on the other hand includes natural language, text, video, and audio. This makes the task of predicting duration of traffic accidents particularly difficult.

Much literature has been published on the factors that influence traffic accidents. Previous studies have shown that an event's time, location, weather, and road conditions significantly impact the duration of a traffic accident. Mohammed et al.^1^ summarizes an analysis of factors affecting the duration of traffic accidents at different stages, data sources, and prediction methods. Zhang et al.^2^ confirms that a larger sample size improves the reliability of the traffic accident duration model. Wali et al.^3^ examined several factors incident durations from more than 45,000 events in Virginia in 2015, including detection source, incident type, road type, and temporal factors. As a summary, these study concluded that response arrival time, accident type, lanes blocked, vehicle type, vehicles involved, deaths and number of rescue vehicles are the main factors affecting the accident duration.

In traffic safety literatures, various modeling techniques have been already utilized to predict the duration of road accidents^4^. Hazard-based modeling is a parametric model, and has been widely utilized in the duration prediction. Nam et al.^5^ applied hazard-based models to evaluate highway incident duration using Two-year data. Chung et al.^6^ developed the log-logistic accelerated failure time (AFT) metric model for temporal stability. Additional studies that utilized hazard-based models for predicting different duration stages include Hojati et al.^7^ and Li et al.^8^ .Moreover, Pang et al.^9^ improved the hazard-based duration modeling using a random parameters with heterogeneity in means and variances approach.

Recently machine learning (ML) is playing an important role in the intelligent transportation. Several comparisons and improvements have been carried out to estimate accident duration. For instance, Restricted Boltzmann Machines^10^, Bayesian Support Vector Regression (BSVR)^11^ ,and the Extreme Gradient Boosting Machine Algorithm (XGBoost)^12^ are evaluated on the prediction of traffic accident duration. Li et al.^13^ established a survival model to deal with the early stage's lack of relevant information about event disposal. Kuang et al.^14^ proposed a two-step model consisting of a Bayesian network and k-nearest neighbor and predicted the duration of accidents using the vehicle sensor data of Xiamen City in 2015. Ghosh et al.^15^ proposed an adaptive ensemble model with traffic speed and flow values from the expressways. As reported the MAPE value of 5–15 min, 16–35 min, 36–200 min, and greater than 200 min, it was observed that Treebagger model outperformed other traditional regression methods. However, the MAPE value is unstable with the range of 20–100.9%. Saracoglu et al.^16^ verified that the prediction accuracy of the decision tree model is about 74%. Hamad et al.^17^ summarized five methods for predicting traffic incident duration by 110,000 incident records with over 52 variables, including Regression Decision Tree, Support Vector Machine (SVM), Ensemble Tree (bagged and boosted), Gaussian Process Regression (GPR), and Artificial Neural Networks (ANN). SVM and GPR models outperformed in terms of accuracy with the best model scoring of MAE is 14.34 min. However, the training time was relatively long from 5 to 34 h. Hamad et al.^18^ utilized an extensive dataset with over 140,000 incident records and 52 variables for predicting incident duration by random forests. The MAE is given in the range of 5–120 min and 1–1440 min respectively. The best MAE is 36.652 min in the wide range. Further, Shang et al.^19^ constructed the hybrid NCA-BOA-RF model to deal with the absence of some feature variables. The results demonstrated high accuracy and robustness. Zhao et al.^20^ built an accident duration prediction model based on heterogeneous ensemble learning with XGBoost, LightGBM, CatBoost, stacking, and an elastic network. Used 2,366,002 initial training set data, the MAPE, MAE, and MSE of the final model are 35.6101%, 30.7432, and 4252.1728, respectively.

Various approaches have been used to solve the challenge of duration prediction for traffic accidents due to the heterogeneity of data and input variables. A growing literature has highlighted the importance of text information on the emergency response to traffic accidents. In addition, event-related information is usually recorded in natural language. Text data in accident reports are considered useful information for understanding accident processes. However, previous studies have often ignored unstructured text information.

Researchers have recently shown increased interest in text data mining on traffic accidents. Text data mostly record relevant information collected from accident investigation, which usually contains rich connotations such as accident characteristics, emergency response measures, and response status. Accordingly, text mining is also often used to identify accident causes and extract useful information. Zhang et al.^21^ analyzed the causes of traffic accidents using the text mining and Latent Dirichlet Allocation algorithms. Ahadh et al.^22^ identified domain-specific keywords and grouped them into topics to analyze accident reports. Zhang et al.^23^ transformed the accident investigation reports data extracts into a labeled dataset for describing events' sequences. Han et al.^24^ analyzed the causation of major traffic accidents based on text mining.

As the improvement of prediction models using structured data is not significant, unstructured data are being collected but underutilized. It is natural to utilize unstructured data to improve traffic accident duration prediction. Pereira et al.^25^ proposed the positive role of text information in predicting the duration of traffic accidents. Sun et al.^26^ improved BERT- BiLSTM-CRF model to extract textual information of traffic accident. Chen et al.^27^ proposed a text-mining-based accident causal classification method based on a relational graph convolutional network (R-GCN) and pre-trained BERT. Using text data, Ji et al.^28^ constructed the V-Fisher ordered clustering model for highway accident duration prediction. Divided into three categories (5–39 min, 40–70 min, and 71–275 min), the accuracy of SVR + LR classification reaches 0.82.

In summary, existing methods for accident duration prediction mostly use machine learning model based on the structured data. Therefore, the literature review revealed the need to compare the different machine learning models for predicting. A few researches have focused on text information from traffic accidents to predict the duration. However, road accident text data usually consists of dozens of high-dimension nonlinear features. Moreover, Current analysis is mostly based on classification models provided by irregular intervals of accident duration. As the heterogeneity of pre-defined categories in the classification models, it is difficult to sort these models with prediction accuracy. Hence, developing text mining techniques to improve road accident duration prediction is difficult. It would be influenced further analysis of traffic accident.

This study utilizes text-based input features combined with traditional accident data to develop a machine learning based accident duration prediction model. The purpose of this study is to better understand text mining in the problem of accident duration prediction. Considering road accidents with textual records, the proposed term frequency-inverse document frequency-random forest (TF-IDF-RF) approach is utilized to analyze the importance of the duration. Furthermore, we can identify significant textual factors for the duration of a traffic accident. Therefore, the results are more interpretable regarding both input and output. Building a heterogeneous model based on text mining and ensemble learning, the model can fully reflect the impact of text mining on prediction while maintaining a good prediction effect.

## Methodology

### Data Collection

The analysis was based on the dataset from China's Shaanxi Province expressway monitoring system. This dataset contains 22,497 samples of traffic accidents in Shaanxi Province from January 2020 to April 2021. Each accident is described by structured data as location, weather, time, and the number of damaged vehicles. At the same time, each accident is recorded and submitted by text in the form of natural language. The records describe the information received and changes in treatment measures. Therefore, each accident may be recorded multiple times. That is, 3138 incidents were recorded in a sample of 22,497 datapoints. The traffic accident duration is calculated from the difference between the system recording time and the accident elimination time. After preprocessing, 3046 traffic accidents were used in this study.

### Traffic accident duration prediction model structure

**Figure 1:**
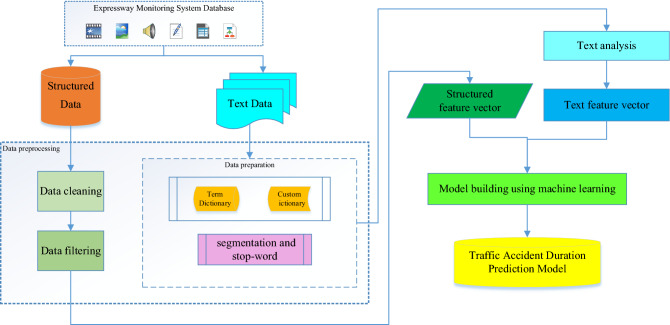
The architecture of traffic accident duration prediction model.

Figure 1 shows the architecture of the duration prediction model for traffic accidents. The research structure and modeling process of this study are shown. Data preprocessing involves filtering and cleaning available data for subsequent modeling and implementation. Data preparation is designed to extract text variables for text-based analysis. It is also important to note that the text data is provided and parsed in Chinese.

### Natural Language Processing

**Figure 2:**
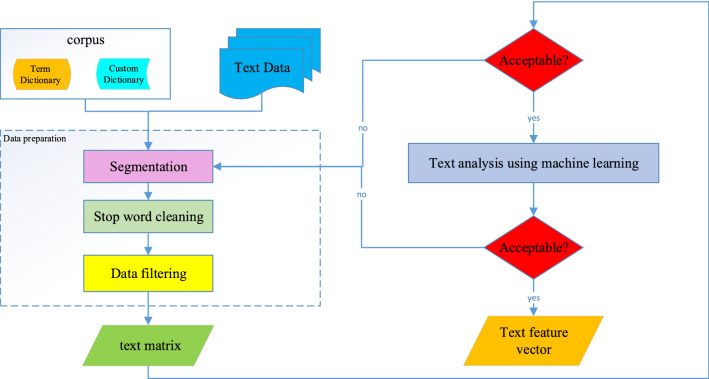
Text data processing phases.

Due to the unstructured data type, the traffic accident text cannot be directly analyzed as a model variable. Natural language processing (NLP) techniques convert the text into feature vectors. Therefore, computers can recognize and process information. The established mathematical model can be used to describe and replace text. Through preprocessing, the text is represented by a multidimensional sparse matrix. Figure 2 shows the text data processing stage.

**Phase 1: Collect transportation terms and build a corpus.**

We first built a corpus (library of words). The corpus comprises common terms, proper names, acronyms, and compound words (e.g., the toll, information board, service area, ascending, inner ring, Xixian, Xiwei, Gao-jiao, and Yanzhiwu).

**Phase 2: Data preparation.**

The pre-processing phase is dedicated to obtaining word vectors. The procedures for text pre-processing are tokenization and stop words removal. With splitting the text into words, punctuation, etc., the unstructured original text of the document is divided into words. We cleaned stop words, concatenated identical words, and removed unwanted, irrelevant words from the analysis.

Firstly, assuming the original texts $$Inf{ = }\left\{ {\begin{array}{*{20}c} {Inf_{1} , \ldots ,Inf_{m} } \\ \end{array} } \right\}$$, a sequence of words is given after text pre-processing by $I = \left\{ {I_{1} , \ldots ,I_{m} } \right\}$,where $I_{i} = \left\{ {s_{i1} ,s_{i2} ,s_{i3} , \ldots ,s_{ih} } \right\}$ is the output for $Inf_{i}$ , *i* = 1 ~ *m* . Then a dataset $W{ = }\left\{ {w_{1} ,w_{2} , \ldots ,w_{n} } \right\}$ is obtained as the union without repeat from $\left\{ {\left\{ {s_{11} ,s_{12} , \ldots ,s_{{1h_{1} }} } \right\}, \ldots ,\left\{ {s_{m1} ,s_{m2} ,s_{{{\text{m3}}}} , \ldots ,s_{{mh_{m} }} } \right\}} \right\}$.

Formally, we can create a text matrix $$A{ = }\left\{ {\begin{array}{*{20}c} {a_{11} } & {a_{12} } & \cdots & {a_{1n} } \\ {a_{21} } & {a_{22} } & \cdots & {a_{2n} } \\ \vdots & \vdots & \vdots & \vdots \\ {a_{m1} } & {a_{m2} } & \cdots & {a_{mn} } \\ \end{array} } \right\}$$ as the feature vector of texts in order to provide a representation of each sample. The mathematical model refers to establishing the matrix *A* to describe texts *I* using feature extraction method.

Term Frequency-Inverse Document Frequency (TF-IDF) is a basic feature extraction method in NLP, and performs very well in terms of the interpretability of results. TF-IDF will not consider the structure or order of words in a document. It estimates the importance of each word in a document based on the weights.

In this paper, the TF-IDF model is used to calculate $a_{ij}$ in mathematical modeling. TF-IDF is a multiplication of TF and IDF. The TF-IDF model can be expressed as $a_{ij} = tf*idf$, where *tf* is denoted as the frequency of the word $w_{j} \in W$ that appears in a corpus $I_{i} = \left\{ {s_{i1} ,s_{i2} , \ldots ,s_{{ih_{i} }} } \right\}$,and *idf* is denoted as the inverse document frequency. When $w_{j} \notin I_{i} = \left\{ {s_{i1} ,s_{i2} , \ldots ,s_{{ih_{i} }} } \right\}$, then $a_{ij} { = 0}$.

**Phase 3: Optimized the results of word segmentation.**

After building a corpus and filtering some unconcerned word repeatability, the results of text pre-processing are constructed and optimized. Finally, the text is transformed from unstructured data into structured data. In other words, with optimizing and updating the dataset $W{ = }\left\{ {w_{1} ,w_{2} , \ldots ,w_{n} } \right\}$ constantly, the n-dimensional matrix *A* is obtained as the feature vector of texts.

**Phase 4: Extract keywords of the text databased on a qualitative analysis.**

The text is characterized by a multidimensional sparse matrix preprocessed and optimized. Since the initial feature space creates a heavy workload for computation and modeling, it is necessary to reduce dimensions to obtain the feature vectors *A*. The feature vectors should not be complex or redundant to distinguish the target text from others. Thus it makes sense to extract key and significant words from the dataset $W{ = }\left\{ {w_{1} ,w_{2} , \ldots ,w_{n} } \right\}$. Assuming the duration as the dependent variable, and the $w_{j} \in W$ as independent variables, the importance degree of each independent variable $w_{j}$ in forecasting is quantified. Then the more important independent variable named keywords are extracted. The method used at this stage will be designed and introduced in details as following.

### The proposed TF-IDF-RF approach

Text data is a rich and special data type. The main characteristics of text data include being (1) super-high-dimension (2), sparse (3), and discrete. Although researchers may be interested in examining the entire data set, it is often more practical to focus on a subsample of data. Specifically, we advocate extracting important words on predicting duration. This study uses Random forest (RF) to extract keywords based on TF-IDF model.

**Figure 3:**
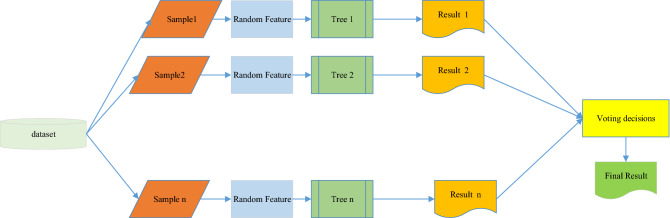
Random forest algorithm.

This section gives a brief overview of RF. Random forest is an ensemble learning, with generating many simple decision trees and aggregating their results^29^. It is a process based on random sampling of data and random selection of features, as described in Fig. 3. Random forest can handle many input variables and can assess the importance of input variables. The algorithm performs well in dealing with computing speed, unbalanced data sets, and missing data. As a regression task, CART (classification and regression tree) is used for the training processes of each base learner.

Besides bootstrap samples, the out-of-bag (OOB) data referred to the un-sampled data or the remaining samples when growing trees. As not included the training data, the out-of-bag (OOB) data can be used as a set of test sample. Each bootstrap sample contains about one-third of the out-of-bag (OOB) data. Therefore, out-of-bag errors (OE) can be used to quantify the model's generalization ability without cross-validation or separate test sets. We can express the out of bag errors (OE) mathematically as:

1 $$OE{ = }\sqrt {\sum\limits_{i = 1}^{u} {\left( {\hat{y}_{i} - y_{i} } \right)^{2} } } ,\quad y_{i} \in D_{oob}$$

where $D_{oob}$ is a set of the out-of-bag (OOB) data, $\hat{y}_{i}$ is predicted value, $y_{i}$ is actual value, *u* is the number of samples in $D_{oob}$.

**Figure 4:**
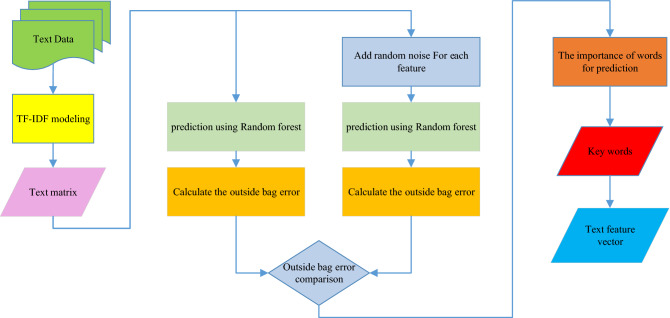
Proposed approach of TF-IDF-RF.

This study uses random forest and text analysis to predict traffic accident duration. In our proposed term frequency-inverse document frequency (TF-IDF) approach, we use the random forest as the basic algorithm to fit the relationship between the text feature vector and traffic accident duration. Keywords are extracted according to the importance of the words in the random forest. Therefore, the TF-IDF of the keywords is calculated as the text feature vector. Further, the text feature vector is optimized by random forest (TF-IDF-RF). The flow chart of TF-IDF-RF is shown in Fig. 4. This is a key contribution of this approach.

**Step 1**: The initial TF-IDF modeling $$TF{ - }idF{ = }\left[ {\begin{array}{*{20}c} {\begin{array}{*{20}c} {t_{11} } & \cdots & {t_{1n} } \\ \vdots & \ddots & \vdots \\ {t_{m1} } & \cdots & {t_{mn} } \\ \end{array} } \\ \end{array} } \right]$$. The TF-IDF model is established as follows.

2 $$TF_{ij} { = }\frac{{n_{ij} }}{{\sum\nolimits_{i}^{{k_{j} }} {n_{ij} } }},$$

3 $$idF_{ij} { = }\frac{N}{{N_{j} + 1}},\,{\text{and}}$$

4 $$t_{ij} = TF_{ij} * idF_{ij} ,$$

where $TF_{ij}$ is described as the term frequency of $w_{i}$ in the sample of $I_{j}$, i = 1 ~ n, j = 1 ~ m. $n_{ij}$ is described as the number of $w_{i}$ in the sample of $I_{j}$, *k*_*j*_ is described as the number of words. $idF_{ij}$ is described as the inverse document frequency, N is the number of samples, and *N*_*j*_ is the number of samples that contain $w_{i}$.

**Step 2**: Superparametric optimization. The number of trees *K* in random forest is a hyperparameter. *K* is not the more the better. In addition, too many trees will offset the advantages brought by the random characteristics. The performance of the model is observed by increasing the number of trees. The best K is selected under an stable performance.

**Step 3**: Training the base learner $Tree_{i}$ in random forest algorithm, *i* = 1 ~ *K.* Thus $Tree_{1} , \ldots ,Tree_{i} , \ldots ,Tree_{K}$ are generated.

**Step 4**: As the input in the random forest algorithm, the matrix $TF{ - }idF$ is used to predict the duration of traffic accidents. The error of OOB is calculated to be OE by the Formula (1).

**Step 5**: Adding the random noise $$\left[ \begin{gathered} \varepsilon_{{1}} \hfill \\ \vdots \hfill \\ \varepsilon_{m} \hfill \\ \end{gathered} \right]$$ for $w_{j}$, then $$TF{ - }idF_{\varepsilon } { = }\left[ {\begin{array}{*{20}c} {t_{11} } \\ \vdots \\ {t_{m1} } \\ \end{array} \begin{array}{*{20}c} \cdots \\ \vdots \\ \cdots \\ \end{array} \begin{array}{*{20}c} {t_{1j} + \varepsilon_{1} } \\ \vdots \\ {t_{mj} + \varepsilon_{m} } \\ \end{array} \begin{array}{*{20}c} \cdots \\ \vdots \\ \cdots \\ \end{array} \begin{array}{*{20}c} {t_{1n} } \\ \vdots \\ {t_{mn} } \\ \end{array} } \right]$$ is used for the input of prediction model. Thus the error of OOB is calculated to be OE_j_.

**Step 6**: When the accuracy is reduced, it is indicated that $w_{j}$ influences the prediction. Thus, $\Delta E_{j} { = }OE - OE_{j}$ reflects the importance of $w_{j}$.

**Step 7**: The top 10% of the words are selected as the keyword set according to importance. Keywords are extracted according to expert knowledge or from the keyword set. Thus, the text feature vector is described as the $TF{ - }idF$ of keywords.

As discussed above, researchers may draw on the computational models as an introductory guide to reduce dimensions for model optimization.

## Results

### Text data analysis

After text data preprocessing, The set of $W{ = }\left\{ {w_{1} ,w_{2} , \ldots ,w_{n} } \right\}$ was extracted, *n* = 3992, including occur, toll station, direction, traffic accident, branch company, highway property, etc.. the matrix *A*_n*1_ is calculated to show the descriptive statistics of a single text. For the text dataset $$Inf{ = }\left\{ {\begin{array}{*{20}c} {I_{1} , \ldots ,I_{m} } \\ \end{array} } \right\}$$, *m* = 3046, the matrix *A*_n*m_ is calculated as features according to the TF-IDF model.

*O*_*tf*_*(j)* is defined as the occurrence number of each word *w*_*j*_ in all texts, which calculated as following formula.

5 $$O_{tf} \left( j \right) = \sum\limits_{i = 1}^{m} {TF_{ij} } ,\quad j = 1\sim n$$

**Figure 5:**
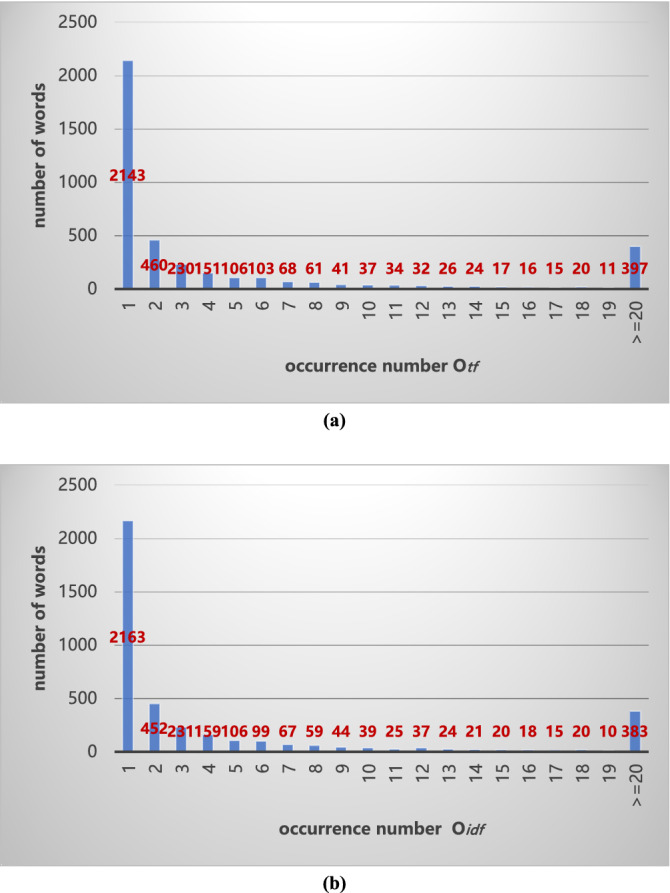
Descriptive statistics of words. (**a**) Term frequency (TF) distribution, (**b**) Document frequency (DF) distribution.

To consistent with the set *W*, a sequence $O_{tf} { = }\left\{ {O_{tf} \left( {1} \right), \ldots ,O_{tf} \left( 2 \right), \ldots ,O_{tf} \left( n \right)} \right\}$ is generated. Histogram statistics is taken in the sequence *O*_*tf*_ to observe the distribution of occurrence number, as shown in Fig. 5a. In consideration of the sparsity and long tail, a group where *O*_*tf*_*(j)* $\ge$ 20 is combinated. In Fig. 5a, the number of words is 2143 that only occur once in all the dataset, and the ratio is 53.68%.

Similarly, a sequence $ND = \left\{ {N_{1} , \ldots ,N_{j} , \ldots ,N_{n} } \right\}$ is generated and analysised by histogram, where *N*_*j*_ is the number of samples that contain *w*_*j*_, *j* = 1 ~ *n*. The results are drawn in Fig. 5b. As shown in Fig. 5b, the number of words is 2163 that only occur once in a document or sample, and the ratio is 54.18%.

It can be summarized that the occurrence number of each word *w*_*j*_ is unbalance and the half of the words are rarely appear. Reducing the dimension of the features matrix is meaningful for efficiency and complexity.

**Figure 6:**
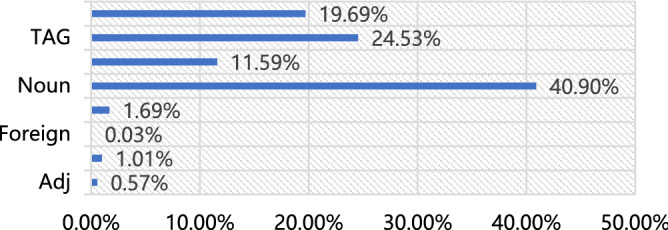
Word frequency by part of speech.

Statistical analysis was conducted according to the parts of speech in Fig. 6. Interestingly, the word frequencies of verbs and the defined corpus is 19.69% and 24.46%, respectively. Thus, the text contains certain emergency response information, and the defined corpus has considerable influence. It is apparent from these figures that many words occur once, and some words occur frequently in each text record.

**Figure 7:**
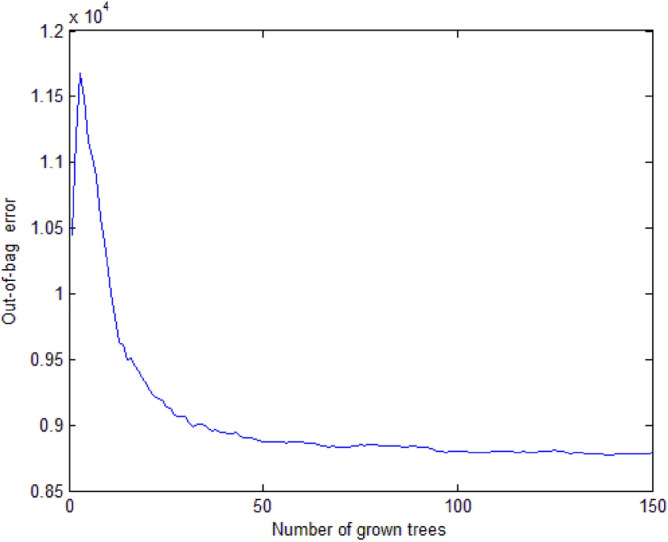
Out of bag error with different numbers of grown trees.

To reduce the vector dimension, the top 10% of 300 words are selected for feature vector representation. Given the tree number of 150, traffic accident duration is predicted based on the random forest by calculating the error in the outside bag. As shown in Fig. 7, it is apparent that the error in the outside bag stays at a certain level with little change when the tree number is more than 50. In consequence, 50 is the most appropriate strategy for the number of trees in random forest.

**Figure 8:**
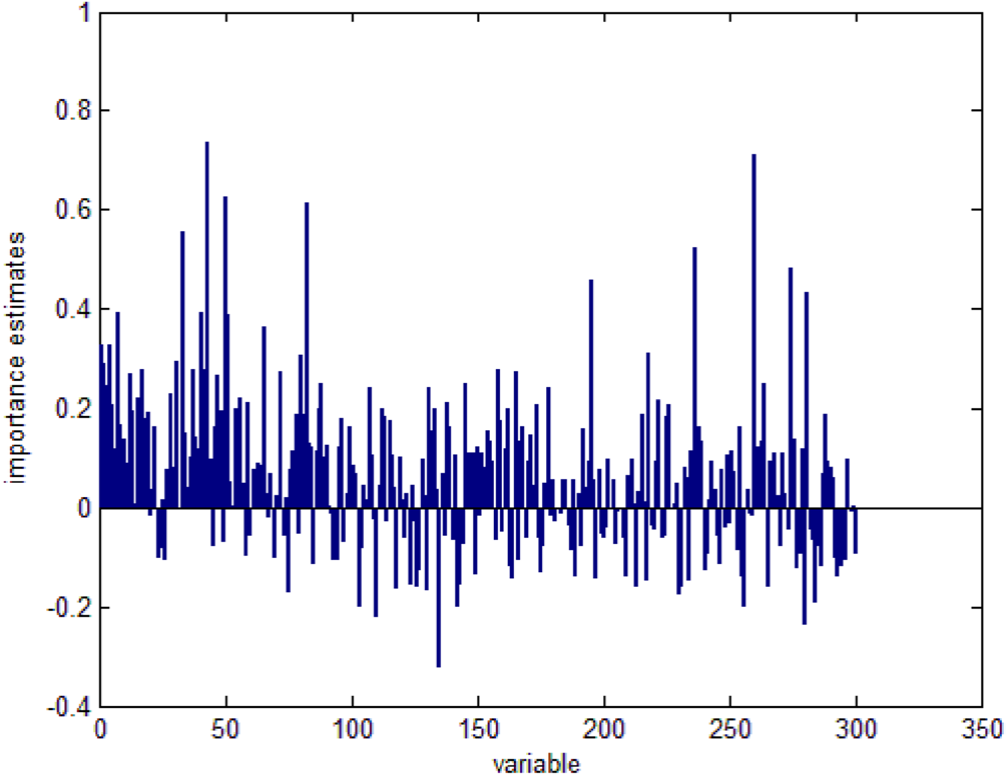
Importance estimates of words.

Under the optimized hyperparameter, the importance of 300 words is analyzed, as seen in Fig. 8. Sorted by importance, the top 5% of the words are constructed as the keyword set *W*_*key*_. As a result, the most significant keywords affecting traffic accident duration include semitrailer, on fire, spontaneous ignition, car, highway property, fire fighting, rollover, trucks, freight, etc.. The number of extraction results is about 50 words. These keywords are meaningful to research traffic accident duration as the important features. More analysis and details of the keywords discussion will be given in the following. The text feature vector is represented as a TF-IDF model of keywords.

### Model comparison

The models were compared to determine the text features' influence on prediction. Namely, the following methods were attempted and compared: decision tree (DT), k nearest neighbor (KNN), support vector regression (SVR), random forest (RF), Gradient Boosting Decision Tree (GDBT), and Xtreme Gradient Boosting (XGBoost). For model comparison in a feature vector, we used three groups of features in the proposed model, as shown in Table 1.

**Table 1:** Feature group.

Variable	Feature group 1	Feature group 2	Feature group 3
**Influence factor**			
Month	√	√	√
Hour	√	√	√
Weekday	√	√	√
Accident type	√	√	√
Accident area	√	√	√
Weather	√	√	√
Location type	√	√	√
Number of injuries	√	√	√
Number of damaged vehicles	√	√	√
Dangerous chemical vehicles	√	√	√
Information board	√	√	√
**Text statistical**			
Report times	**╳**	√	√
Text size of first report	**╳**	√	√
**Text content**			
Keywords TF-IDF vector	**╳**	**╳**	√

RMSE (root mean square error), MAPE (mean absolute percentage error), and R^2^ were used to measure the prediction effect. Some machine learning algorithms were compared, including DT, KNN, SVR, RF, GDBT, and XGBoost. Table 2 shows the prediction effect through quantitative indicators in the training and test sets.

**Table 2:** Prediction results of different algorithms for each feature group.

Model	Dataset	Feature group 1			Feature group 2			Feature group 3		
		RMSE	MAPE	R^2^	RMSE	MAPE	R^2^	RMSE	MAPE	R^2^
DT	Train	74.158	63.921	0.403	60.501	60.306	0.602	57.69	58.216	0.638
	Test	118.896	86.899	− 0.248	110.969	79.508	− 0.087	101.108	79.874	0.098
KNN	Train	83.716	59.104	0.239	79.37	58.49	0.316	79.557	58.62	0.312
	Test	111.303	103.463	− 0.094	111.384	101.168	− 0.095	111.612	101.89	− 0.1
SVR	Train	97.512	125.527	− 0.033	102.542	992.803	− 0.142	117.241	2905.874	− 0.493
	Test	113.733	218.901	− 0.142	117.079	1383.099	− 0.21	130.378	774.661	− 0.501
RF	Train	66.868	59.042	0.514	61.87	56.197	0.584	54.372	51.566	0.679
	Test	106.59	75.258	− 0.003	98.606	75.274	0.142	94.839	73.235	0.206
GDBT	Train	39.712	41.961	0.829	27.258	34.368	0.919	19.378	28.269	0.959
	Test	118.187	99.109	− 0.233	102.064	81.054	0.08	94.437	79.585	0.213
XGBoost	Train	30.016	29.882	0.902	14.48	18.751	0.977	15.988	22.502	0.972
	Test	121.691	130.977	− 0.307	108.459	97.432	− 0.038	99.159	80.927	0.132

**Figure 9:**
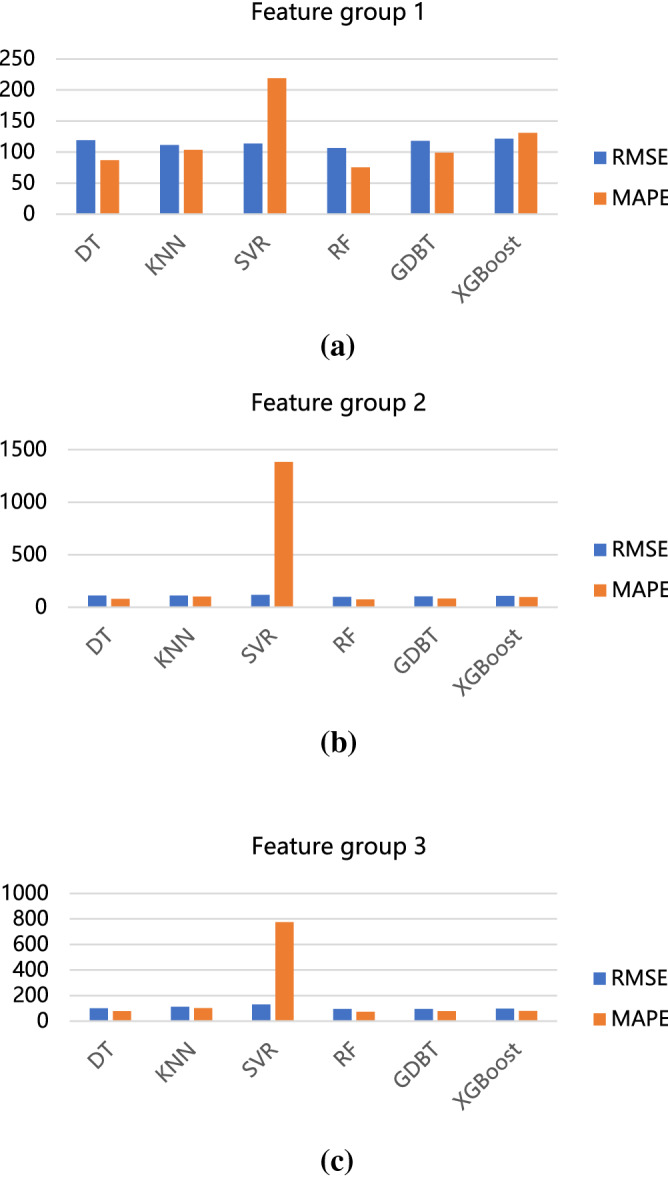
Comparison of model errors with RMSE and MAPE. (**a**) The input variable of feature group 1, (**b**) The input variable of feature group 2, (**c**) The input variable of feature group 3.

**Figure 10:**
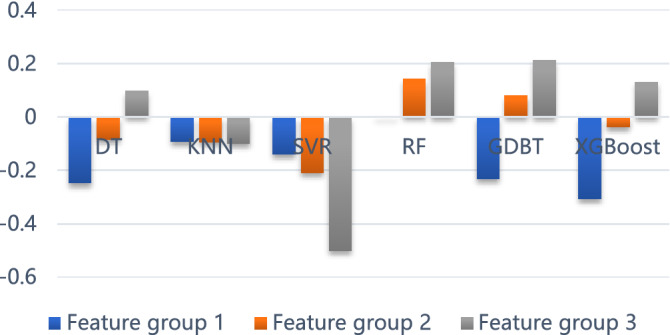
The R^2^ of models at different feature groups.

Figure 9 shows the RMSE and MAPE of models under different groups of input variables. When comparing different feature groups, the prediction performance is slightly improved for all machine learning algorithms by adding texts in the input features. With lower forecast errors in RMSE and MAPE, it is apparent that the random forest algorithm offers the best prediction effect. The results on R^2^ are presented in Fig. 10. Notably, the random forest matches the forecast results well.

Overall, our analysis highlighted features that can be useful in practice. From the numerical analysis results discussed above, we conclude that the text feature vector can be utilized in traffic accident duration prediction for more accurate results. Calculations have shown that the random forest is an effective tool.

### Model optimization

The effect of model input variables on the predicted results of the lightweight model is discussed in application.

Random forest is used to analyze the importance of the features, as presented in Table 3. The model input is simplified according to the importance ranking under approximate predictive effect. Model inputs are grouped by cumulative importance from 30 to 70%. The five groups were 31.10%, 40.50%, 51.00%, 60.20%, and 70.90%.

**Table 3:** Results of feature importance analysis.

Variable	Importance (%)	Cumulative percent (%)	Model input 1	Model input 2	Model input 3	Model input 4	Model input 5
Report times	17.80	17.80	√	√	√	√	√
Hour	13.30	**31.10**	√	√	√	√	√
Text size of first report	3.50	34.60	**╳**	√	√	√	√
W_1_	3.00	37.60	**╳**	√	√	√	√
W_2_	2.90	**40.50**	**╳**	√	√	√	√
W_3_	2.40	42.90	**╳**	**╳**	√	√	√
Mouth	2.30	45.20	**╳**	**╳**	√	√	√
W_4_	2.10	47.30	**╳**	**╳**	√	√	√
W_5_	1.90	49.20	**╳**	**╳**	√	√	√
Accident type	1.80	**51.00**	**╳**	**╳**	√	√	√
W_6_	1.80	52.80	**╳**	**╳**	**╳**	√	√
W_7_	1.60	54.40	**╳**	**╳**	**╳**	√	√
W_8_	1.60	56.00	**╳**	**╳**	**╳**	√	√
W_9_	1.40	57.40	**╳**	**╳**	**╳**	√	√
W_10_	1.40	58.80	**╳**	**╳**	**╳**	√	√
W_11_	1.40	**60.20**	**╳**	**╳**	**╳**	√	√
W_12_	1.30	61.50	**╳**	**╳**	**╳**	**╳**	√
Weather	1.20	62.70	**╳**	**╳**	**╳**	**╳**	√
W_13_	1.10	63.80	**╳**	**╳**	**╳**	**╳**	√
W_14_	1.00	64.80	**╳**	**╳**	**╳**	**╳**	√
Weekday	0.90	65.70	**╳**	**╳**	**╳**	**╳**	√
W_15_	0.90	66.60	**╳**	**╳**	**╳**	**╳**	√
W_16_	0.90	67.50	**╳**	**╳**	**╳**	**╳**	√
W_17_	0.90	68.40	**╳**	**╳**	**╳**	**╳**	√
W_18_	0.90	69.30	**╳**	**╳**	**╳**	**╳**	√
W_19_	0.80	70.10	**╳**	**╳**	**╳**	**╳**	√
W_20_	0.80	**70.90**	**╳**	**╳**	**╳**	**╳**	√
W_21_	0.80	71.70	**╳**	**╳**	**╳**	**╳**	**╳**

Significant values are in bold.

**Figure 11:**
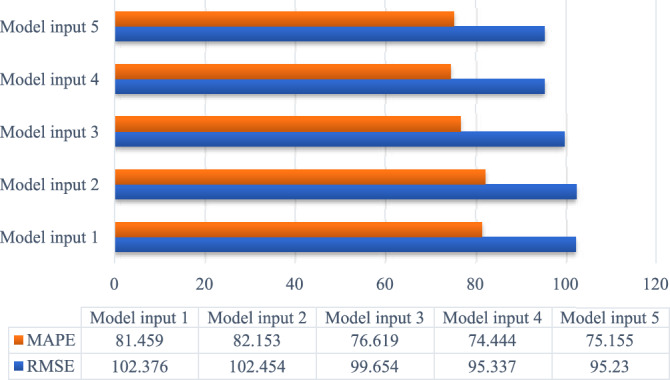
Comparison of prediction effects under different model inputs.

**Figure 12:**
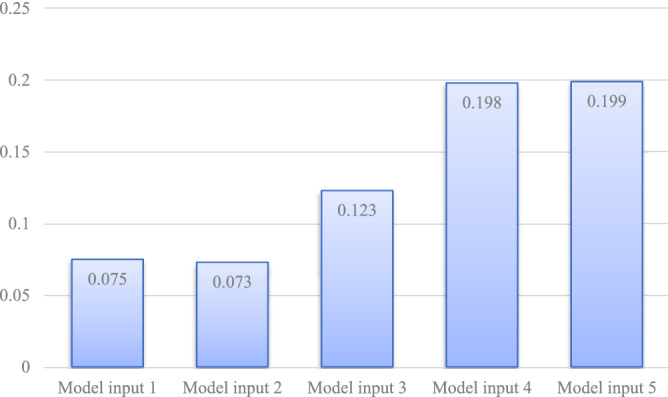
Comparison of R^2^ under different model inputs.

The results of comparing MAPE and RMSE are shown in Fig. 11. As seen in Fig. 11, the MAPE and RMSE values gradually decrease as the dimensionality of input variables increases. A more detailed illustration of R^2^ is in Fig. 12. The more input variables there are, the higher R^2^.

However, when the cumulative importance reaches 60%, the predicted indexes no longer increase significantly. The cumulative importance of 60% is sufficient for controlling input dimensions. By simplifying the input variables of the model, we obtained superior results.

## Discussion

This paper proposes the feature vector fortext data by a hybrid model, TF-IDF-RF. We compare the model in different input groups with base learner models: DT, KNN, SVR, RF, GDBT, and XGBoost. Based on a dataset of traffic accidents, we study text features in the accident duration prediction problem and construct a model of accident duration prediction based on heterogeneous data. The model performs well and combines the advantages of multiple input features, which results are more comprehensive. The results show that report times, hour, and text size of first report are important to the accident duration.

One of the aims of this study was to compare the performance of base learner model for predicting traffic incident duration. The most obvious finding to emerge from the analysis is that RF performs better than other models in terms of RMSE, MAPE, and R^2^, as shown in Table 2. Consistent with the literatures, this research found the important roles of ensemble algorithm in accident duration prediction. This indicates the superiority of RF in predicting traffic accident duration.

To our knowledge there are only a few reports in the literature with detailed analysis of text-based duration prediction. In this study, we set out to assess the importance of textual data in the accident duration prediction. It is interesting to compare prediction models with different input group in Table 2. The most remarkable result to emerge from the data is that RF with feature group 3 performs better than other input groups. This suggests that the text feature vectors can significantly improve the prediction performance of RF. Significantly, keywords were found to be able to influence the results of duration prediction. We believe that the result emphasizes the validity of our model.

As mentioned in the previous literatures, the MAPE value is in a wide range of variation. For example, 20–100% was also reported by Ghosh et al., and 56–184% was also reported by Li et al.. Compared with regression models, the MAPE value of our study is stable within 73–75%. Moreover, the fusion of different heterogeneous data will help to give consideration to the accuracy and stability of the results. Therefore, our study is more like a balance between accuracy, efficiency and interpretability.

Several reports have shown that various factors affect accident duration. The present study was designed to determine the effect of identified features from textual data. The diversity of keywords has provide a meaningful understanding with different topics. There are several possible explanations for this result. According to the keywords related to vehicle type, we infer that it is necessary to pay attention to semitrailer, car, truck, and bus. Our findings appear to be well substantiated that rollover and crash are the important input feature in accident type. The findings reported here suggest that fire fighting, highways, traffic police, and branches are the important feature affecting traffic accident duration in terms of participating institutions. Surprisingly, the research was successful as it was able to identify emergency disposal, such as wait, rush, follow-up report, etc. As far as we know, this is the most critical aspects of accident duration. Ultimately, such information will beneficially help in mitigating traffic congestion due to accidents.

It is crucial to note that the importance of input features under the fusion of structured data and text. Further researches were carried out to prove their advantages in modeling. Table 3 lists the importance of input features and cumulative percentage for traffic accident duration. We found that the first report's times, hours, and text size are the most important explanatory factors of traffic accident duration. Moreover, the most significant keywords affecting traffic accident duration in the text are extracted. As expected, our experiments support the work of other studies in this area linking textual data with traffic accident duration. The training speed of our model is significantly higher than that reported by Hamad et al. The prediction model based on regression provides convenience for the application. The model achieves higher accuracy and has good interpretability. Thus, the text dataset significantly affects the performance of the model. Regrettably, due to the limited sample size and the complexity of traffic accidents, it is inevitable to appreciate that short-range forecasting was below expectations.

In summary, the present results are significant in two major respects. Firstly, the hybrid TF-IDF-RF improves the prediction performance and provides interpretable results. Secondly, some measures are put forward, such as paying attention to the road, communication text, and formulating corresponding text requirements. A further study with more focus on textual descriptions of the disposal processes is therefore suggested.

## Conclusions

Predicting traffic accident duration is important for accident management. As unstructured data, text information is often ignored. The author has proposed a machine learning approach to predict the duration of accidents on expressways. The present study finds that some textual features are more important for duration prediction than others.

This study proposes a hybrid TF-IDF-RF model for textual feature extraction in traffic accidents. Experimental results show that the TF-IDF-RF model has higher prediction accuracy than traditional machine learning models and simplifies the model variables. Understanding the model helps identify the main influencing factors in traffic accident text. The search results provide references for documenting transit text.

## Data Availability

The data used in this study are available from the corresponding author upon request.

## References

[1] Mohammed, Z. A., Abdullah, M. N. & Al-Hussaini, I. H. Review of the traffic incident duration prediction methods. *J. Res. Sci. Eng.* **2**(6) (2020).

[2] Zhang Z, Liu J, Li X, Khattak AJ (2021). Do larger sample sizes increase the reliability of traffic incident duration models? A case study of east Tennessee incidents. Transp. Res. Rec..

[3] Wali B, Khattak AJ, Liu J (2021). Heterogeneity assessment in incident duration modelling: Implications for development of practical strategies for small & large scale incidents. J. Intell. Transp. Syst..

[4] Yuan H, Li G (2021). A survey of traffic prediction: From spatio-temporal data to intelligent transportation. Data Sci. Eng..

[5] Nam D, Mannering F (2000). An exploratory hazard-based analysis of highway incident duration. Transp. Res. Part A.

[6] Chung Y (2009). Development of an accident duration prediction model on the Korean Freeway Systems. Accid. Anal. Prev..

[7] Hojati AT, Ferreira L, Washington S, Charles P (2013). Hazard based models for freeway traffic incident duration. Accid. Anal. Prev..

[8] Li R, Guo M, Lu H (2017). Analysis of the different duration stages of accidents with hazard-based model. Int. J. Intell. Transp. Syst. Res..

[9] Pang J, Krathaus A, Benedyk I, Ahmed SS, Anastasopoulos PC (2022). A temporal instability analysis of environmental factors affecting accident occurrences during snow events: The random parameters hazard-based duration model with means and variances heterogeneity. Anal. Methods Accid. Res..

[10] Li LC, Sheng X, Du BW, Wang YG (2020). A deep fusion model based on restricted Boltzmann machines for traffic accident duration prediction. Eng. Appl. Artif. Intell..

[11] Ghosh B, Dauwels J (2021). Comparison of different Bayesian methods for estimating error bars with incident duration prediction. J. Intell. Transp. Syst..

[12] Tang JJ, Zheng LL, Han CY (2020). Traffic incident clearance time prediction and influencing factor analysis using extreme gradient boosting model. J. Adv. Transp..

[13] Li X, Liu J, Khattak A (2020). Sequential prediction for large-scale traffic incident duration: Application and comparison of survival models. Transp. Res. Rec..

[14] Kuang L, Yan H, Zhu YJ (2019). Predicting duration of traffic accidents based on cost-sensitive Bayesian network and weighted K-nearest neighbor. J. Intell. Transp. Syst..

[15] Ghosh B, Asif MT, Dauwels J (2018). Dynamic prediction of the incident duration using adaptive feature set. IEEE Trans. Intell. Transp. Syst..

[16] Saracoglu A, Ozen H (2020). Estimation of traffic incident duration: A comparative study of decision tree models. Arab. J. Sci. Eng..

[17] Hamad K, Al-Ruzouq R, Zeiada W (2020). Predicting incident duration using random forests. Transp. A Transp. Sci..

[18] Hamad K, Khalil MA, Alozi AR (2019). Predicting freeway incident duration using machine learning. Int. J. Intell. Transp. Syst. Res..

[19] Shang Q, Tan D, Gao S, Feng L, Khazaei H (2019). A hybrid method for traffic incident duration prediction using BOA-optimized random forest combined with neighborhood components analysis. J. Adv. Transp..

[20] Zhao YX, Deng W (2022). Prediction in traffic accident duration based on heterogeneous ensemble learning. Appl. Artif. Intell..

[21] Zhang JH, Shao KJ, Guan TC (2019). Application of traffic environment accident information text processing technology based on LDA topic model. Ekoloji.

[22] Ahadh A, Binish GV, Srinivasan R (2021). Text mining of accident reports using semi-supervised keyword extraction and topic modeling. Process Saf. Environ. Prot..

[23] Zhang XG, Srinivasan P, Mahadevan S (2021). Sequential deep learning from NTSB reports for aviation safety prognosis. Saf. Sci..

[24] Han TY (2021). Network analysis on causes for serious traffic accidents based on text mining. China Saf. Sci. J..

[25] Pereira FC (2013). Text analysis in incident duration prediction. Transp. Res. Part C.

[26] Sun H (2021). Traffic Accident Text Analysis Based on BERT+Bi LSTM+CRF Model and Improved Apriori Algorithm.

[27] Chen ZL, Huang K, Wu L, Zhong ZY, Jiao ZY (2022). Relational graph convolutional network for text-mining-based accident causal classification. Appl. Sci..

[28] Ji KK (2020). A predictive model of highway accident duration driven by text data. Traffic Inf. Saf..

[29] Hastie, T, Tibshirani, R. & Friedman, J. Unsupervised learning. In *The Elements of Statistical Learning*. (Springer, 2009).

